# Annotations

**Published:** 1907-07-13

**Authors:** 


### Annotations.

### Sir James Barr on Physical Deterioration.

Sir James Barr is nothing if not robust and thorough, and his recent address, as president of the preventive medicine section of the Royal Institute of Public Health, gives us further evidence of the qualities to which previous experience of his utterances had introduced us. In vigorous fashion Sir James criticised the work of the sanitarians, whose comparative failure he attributed to a too exclusive attention to the prevention of zymotic diseases and to the preservation of life. The ambition of sanitarians and medical practitioners alike should be the improvement of health, and the public, were they alive to their own interests, would pay their medical advisers for directing them in the ways of health rather than for mere attendance and advice during the presence of disease. There is a great deal of ill-health which deserves not sympathy or pity, but criticism and condemnation, and a worship of good health, including works as well as faith, might with advantage be made a feature of the national religion. To return to Sir James Barr's address, we find he would encourage from early youth upwards the best means of developing the moral and physical grit of the nation. Every child under sixteen years of age should as far as necessary be fed and cared for by the State. The aged should be supported by a tax placed on multi-millionaires. The decline in the birth-rate and the high death-rate in early infancy are both capable of being largely met by State interference. Our sailors should be encouraged to marry, and suitable provision should be made for their wives and children. These—and they do not exhaust the catalogue—are vigorous proposals, but, details apart, it will be recognised that their boldness carries the vote of the earnest and thoughtful reformer.

### The Society of Tropical Medicine and Hygiene.

Sir Patrick Manson, in delivering his inaugural address as President of this newly founded Society, gave a most interesting and instructive sketch of the wonderful advances made in the investigation of tropical diseases during recent years. Certainly the record which will have one day to be added to the history of medicine will mark the later part of the nineteenth century as the date of the commencement of a great and striking development. Even in an authoritative work published in 1893, the chapter on malaria contained not a single word on the mosquito as an agent in the diffusion of the disease. Now that the mosquito is recognised as the sole medium by which distribution is effected, and an enormous stimulus has been given to the study of the part played by insects in the carriage of disease germs. In reference to sleeping sickness, this is recognised as the terminal phase of a trypanosoma infection, and the cause of the sleep is known to be an infiltration of the lymphatic spaces of the brain by certain small mononuclear cells; in addition, it is known that the tsetse fly conveys the disease, and that arsenic, mercury, and certain dyes exercise some degree of control over the symptoms. Another advance has been the discovery that the larval ankylostome obtains access to the intestinal canal by penetrating the skin. As indicating the important part played by them in the spread of disease, Sir Patrick stated it is necessary to have knowledge of some 600 species of mosquitoes, not to mention the ticks, tsetse flies, and other less important blood-suckers. Attention was also drawn to the encouraging attitude adopted by the British and other Governments towards work in tropical medicine, to the value of international conferences, and to the need for a society such as that now founded, by which further progress could be both advanced and communicated.

### Guy's Hospital Medical School.

The annual distribution of prizes to the students of this school took place on the 6th inst., when Mr. Cosmo Bonsor, the Treasurer of the Hospital, presided. The report of the Dean (Mr. H. L. Eason) presented a number of gratifying features. Especially is it satisfactory to note that the entry of students for the academic year was the highest for the last thirteen years, the entrants for the full curriculum numbering ninety-two. Another announcement of interest was that relating to the pathological department. A vacancy having occurred in the lectureship in pathology, it has been determined to reorder the conditions of the appointment, and to arrange that the new lecturer shall give the whole of his time to the teaching and laboratory work. This, it will be recognised, is a step in the right direction. The demands of modern pathology and the increased association of the subject with clinical work render it imperative, if the routine work is to be adequately discharged, and still more if original observation is to be developed, that the officer in charge shall give his undivided attention to his duties. Guy's is fortunate in possessing an endowed lectureship of experimental pathology, and this has been generously placed at the service of the school by the founder, Mr. Robert Gordon. With this help the Treasurer has been able to establish a Gordon Lectureship of Pathology, to which a salary of £500 per annum will be attached. In view of the sums paid in some of the non-Metropolitan schools for similar duties the income cannot be called excessive. Still, it is a distinct advance, and we cordially congratulate the authorities on this development in the school. Attention is also drawn in the Report to the fact that though the curriculum has become both longer and more complex the inclusive fee remains without change. To advance that fee is recognised to be impossible, because the average income to be made from the practice of medicine does not justify it. That, indeed, is an aspect of medical education which cannot be neglected. And sooner or later it will carry consequences for the public. For it is hopeless to contend against economic forces, and unless a more adequate return is possible a certain proportion of those who propose for themselves a medical career will be deflected into other callings.

